# Heart Rate Variability and Recurrent Stroke and Myocardial Infarction in Patients With Acute Mild to Moderate Stroke

**DOI:** 10.3389/fneur.2021.772674

**Published:** 2021-12-23

**Authors:** Regina von Rennenberg, Thomas Krause, Juliane Herm, Simon Hellwig, Jan F. Scheitz, Matthias Endres, Karl Georg Haeusler, Christian H. Nolte

**Affiliations:** ^1^Klinik und Hochschulambulanz für Neurologie, Charité – Universitätsmedizin Berlin, Corporate Member of Freie Universität Berlin, Humboldt-Universität zu Berlin, and Berlin Institute of Health (BIH), Berlin, Germany; ^2^Center for Stroke Research Berlin (CSB), Charité – Universitätsmedizin Berlin, Berlin, Germany; ^3^German Center for Neurodegenerative Diseases (Deutsches Zentrum für Neurodegenerative Erkrankungen), Berlin, Germany; ^4^Department of Neurology, Jüdisches Krankenhaus Berlin, Berlin, Germany; ^5^German Center for Cardiovascular Research (Deutsches Zentrum für Herz-Kreislaufforschung), Berlin, Germany; ^6^Department of Neurology, Universitätsklinikum Würzburg, Würzburg, Germany

**Keywords:** stroke, heart rate variability, functional outcome, cardiovascular events, heart and brain interaction

## Abstract

**Objectives:** In patients with acute ischemic stroke, reduced heart rate variability (HRV) may indicate poor outcome. We tested whether HRV in the acute phase of stroke is associated with higher rates of mortality, recurrent stroke, myocardial infarction (MI) or functional outcome.

**Materials and Methods:** Patients with acute mild to moderate ischemic stroke without known atrial fibrillation were prospectively enrolled to the investigator-initiated Heart and Brain interfaces in Acute Ischemic Stroke (HEBRAS) study (NCT 02142413). HRV parameters were assessed during the in-hospital stay using a 10-min section of each patient's ECG recording at day- and nighttime, calculating time and frequency domain HRV parameters. Frequency of a combined endpoint of recurrent stroke, MI or death of any cause and the respective individual events were assessed 12 months after the index stroke. Patients' functional outcome was measured by the modified Rankin Scale (mRS) at 12 months.

**Results:** We included 308 patients (37% female, median NIHSS = 2 on admission, median age 69 years). Complete follow-up was achieved in 286/308 (93%) patients. At 12 months, 32 (9.5%), 5 (1.7%) and 13 (3.7%) patients had suffered a recurrent stroke, MI or death, respectively. After adjustment for age, sex, stroke severity and vascular risk factors, there was no significant association between HRV and recurrent stroke, MI, death or the combined endpoint. We did not find a significant impact of HRV on a mRS ≥ 2 12 months after the index stroke.

**Conclusion:** HRV did not predict recurrent vascular events in patients with acute mild to moderate ischemic stroke.

## Introduction

The state of the autonomic nervous system can be assessed by heart rate variability (HRV) using long term (i.e. 24 h) or short term (i.e. 5–10 min) Holter ECG (1). To date, there is no universal recommendation which HRV parameters are best to examine the autonomic nervous system. One can calculate time domain (differences in beat-to-beat intervals) and frequency domain (spectral components of the tachogram) parameters (1).

Large prospective studies conducted in the general population have shown an association between HRV and incident stroke (2, 3). In hypertensive patients, reduced HRV was associated with an increased risk of stroke as well as acute coronary syndrome (ACS) (4). Moreover, previous studies have reported that HRV is reduced in acute ischemic stroke patients compared to healthy controls (5–7). Compared to stroke patients with normal HRV, those with reduced HRV showed increased mortality and poor functional outcome in several retrospective and prospective studies (5, 8–10). However, these studies were based on <100 stroke patients and data on the association between HRV and adverse vascular events such as recurrent stroke or myocardial infarction (MI) are lacking in stroke patients. Furthermore, the exact mechanism by which reduced HRV leads to poor outcome in stroke patients is unknown (6, 8, 9). There is evidence that ischemic brain injury itself may induce dysregulation of the central autonomic network, which may subsequently lead to myocardial injury and even myocardial infarction (11, 12). On the other hand, reduced HRV may also be present before stroke, indicating a pre-existing stroke risk factor. Whatever the exact mechanism, dysregulation of the autonomic nervous system may contribute to risk of recurrent stroke and MI.

In this cross-sectional study, we examined the impact of HRV parameters as predictors of recurrent stroke, MI, mortality as well as on functional outcome in patients with acute ischemic stroke.

## Materials and Methods

### Study Design and Study Population

Here, we report a secondary outcome measure of the HEart and BRain Interfaces in Acute Ischemic Stroke (HEBRAS) study (NCT02142413). The protocol of the investigator-initiated, cross-sectional, single-center study has been published elsewhere (13). The HEBRAS study aimed to assess whether a more enhanced diagnostic work-up including cardiac MRI, MR-angiography, and prolonged Holter ECG monitoring leads to a higher detection rate of pathologic findings relevant to determine stroke etiology compared to routine diagnostic work-up. Patients with acute ischemic stroke confirmed by cerebral imaging (CT or MRI) and no history of atrial fibrillation were enrolled at the Charité-University Hospital, Campus Benjamin Franklin, Berlin, Germany within six days after stroke onset. Patients with contraindications to MRI, renal failure or severe heart failure (NYHA III or IV) were not included. The HEBRAS study conforms with the World Medical Association Declaration of Helsinki and was approved by the local ethics committee of Charité-Universitätsmedizin Berlin (EA2/033/14). All patients included provided written informed consent. The data that support the findings of this study are available from the corresponding author upon reasonable request.

### Data Assessment

We acquired information on patients' medical history and current medication as well as clinical status during the baseline visit in hospital. The National Institutes of Health Stroke Scale (NIHSS) was used to determine clinical stroke severity (14). The modified Rankin Scale (mRS) was used to assess patients' functional outcome (15).

Holter ECG recording was performed after enrolment for up to 10 days after hospital discharge using the portable CardioMem 4000 (GETEMED AG, Teltow, Germany). We measured HRV using a 10-min section of each patient's ECG recording. As the sympathetic and parasympathetic influence are known to vary during the course of the day, we examined one section during daytime (6 p.m. ± 1 h) and one section during nighttime (3 a.m. ± 1 h) using the earliest available recording for each patient. Automatic beat classification was verified and corrected appropriately by a trained physician (RR). We excluded patients whose Holter ECG recordings did not fulfill data quality criteria for HRV analysis, i.e., who were not in sinus rhythm or had ≥ 20% ectopic beats (1).

We calculated time domain as well as frequency domain measurements using the GETEMED software CardioDay V2.5. According to the guidelines established by the European Society of Cardiology and the North American Society of Pacing Electrophysiology, the standard deviation of beat-to-beat (NN) intervals (SDNN) is considered as a parameter of the overall HRV, whereas the Root Mean Square of Successive Differences (RMSSD) is an estimate of the short-term components of HRV (1). The low frequency power component (LF) covers the frequency range of 0.04–0.15 Hertz (Hz) and is considered to include both sympathetic and vagal modulation (1). The high frequency power component (HF) with a frequency range of 0.15–0.4 Hz is assumed to be a marker of the efferent vagal activity, whereas the LF/HF ratio is regarded as a measurement of the efferent sympathetic activity (1). We measured LF and HF in normalized units (nu), which represent the relative value of each component in proportion to the total power (2). This diminishes the effect of changes in the total power on the values of LF and HF.

### Follow-Up

Patients were followed-up by a standardized telephone interview three and 12 months after the index stroke and were questioned about the occurrence of adverse vascular events (i.e., recurrent ischemic stroke according to the WHO definition or MI). Functional outcome according to mRS was assessed (16). A mRS score ≥ 2 was defined as poor functional outcome. In case of no response, we obtained information on patients' vital status from the local registration office.

Follow-up interviews were conducted blinded to clinical information, imaging and investigation findings.

### Statistical Analysis

Univariate analyses of associations between baseline characteristics and clinical outcome were performed using Chi-Square test for binomial and Mann-Whitney U test for continuous variables since data were non-normally distributed. For the association between baseline characteristics and HRV parameters, we used Mann-Whitney U test for binomially scaled and Correlation analysis for continuously scaled baseline characteristics.

We performed logistic regression analysis to assess the effect of HRV on recurrent stroke, MI and death. Moreover, we tested the possible association between HRV parameters and the composite endpoint of stroke, MI and death (MACE) as well as poor functional outcome. For the assessment of the combined endpoint, patients were censored after reaching one of the respective endpoints. Logistic regression was performed according to three different models. After running an unadjusted model, we included age (continuous) and sex (dichotomous) as covariates. In the third model, we additionally adjusted for baseline stroke severity (NIHSS; continuous), history of diabetes (dichotomous), history of coronary artery disease (dichotomous), medication with beta-blockers on admission (dichotomous), medication with inhibitors of the renin-angiotensin-aldosterone system (RAAS) on admission (dichotomous) and medication with antidepressants on admission. In addition, we performed a subgroup analysis excluding all patients medicated with beta-blockers, RAAS inhibitors or antidepressants on admission and re-ran the logistic regression analyses according to the three adjustment models.

We performed all statistical calculations at a 0.05 significance level using SPSS Statistics 23.0 (IBM, Armonk, NY). We made no adjustment for multiple comparisons.

## Results

### Baseline Characteristics

**Figure 1:**
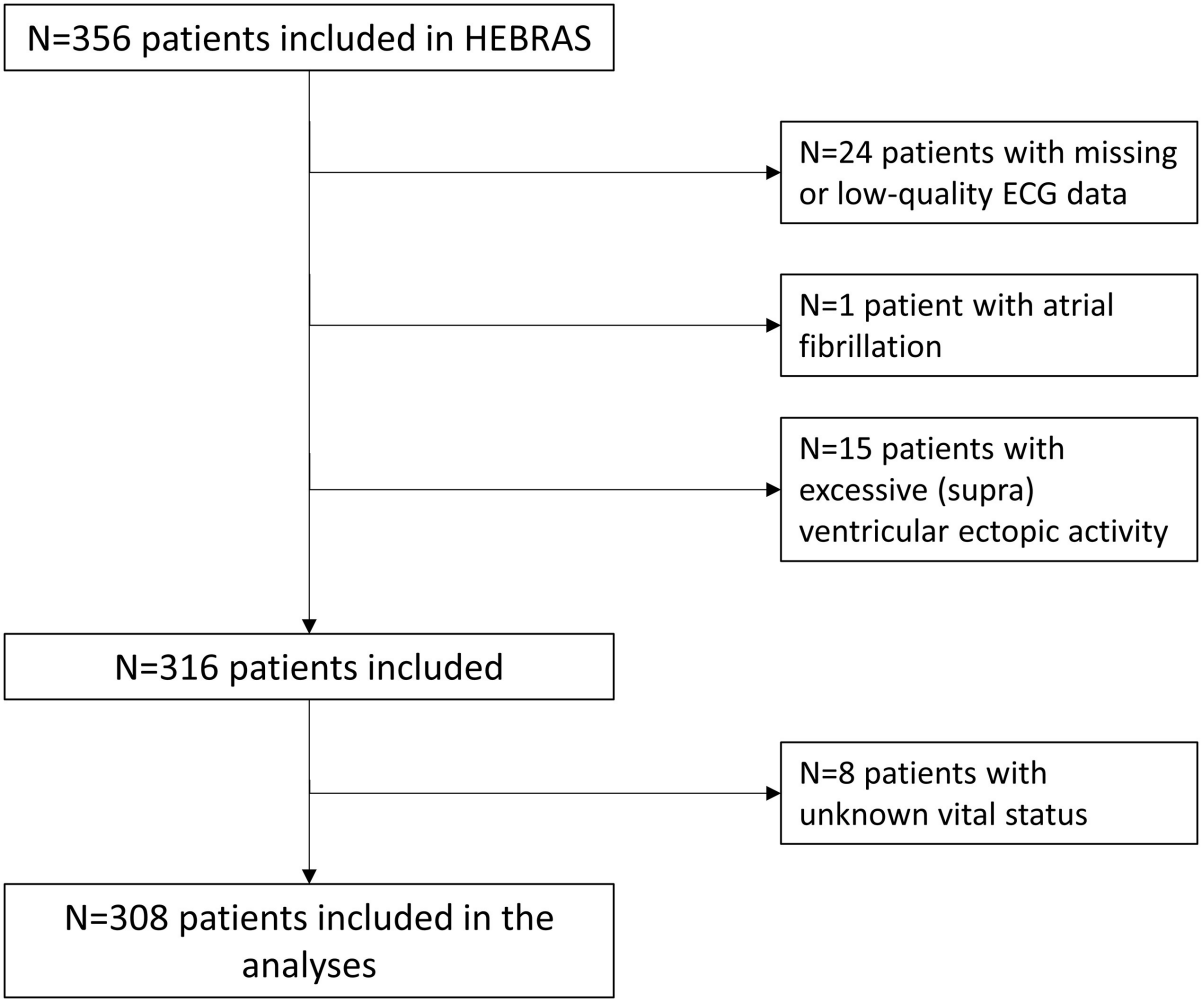
Algorithm for inclusion/exclusion of patients.

Patients' baseline characteristics and median HRV parameters are displayed in Table 1. We had to exclude 40 patients (11.2%) due to missing or low-quality ECG data (*n* = 24), AF (*n* = 1) or excessive (supra)ventricular ectopic activity (*n* = 15) rendering HRV analysis impossible (see also Figure 1). No follow-up information was available in 11 (3.5%) patients after three months and 27 (8.5%) patients after one year. Of these, we could obtain information on vital status from registration offices in 19 patients, resulting in known vital status in 308 (97.6%) patients that were included in the analysis. Median age of 308 study patients was 69 (IQR 58–75) years, 63% were male and median NIHSS at baseline was 2 (IQR 1–4).

**Table 1:** Baseline characteristics and HRV parameters of 308 HEBRAS patients included in the analysis.

**Baseline characteristics**	
Age, median (IQR)	69 (58–75)
Female Sex, n (%)	117 (37.0%)
Initial NIHSS	2 (1–4)
History of hypertension, n (%)	183 (57.9%)
History of diabetes, n (%)	64 (20.3%)
History of coronary artery disease, n (%)	30 (9.5%)
History of heart failure, n (%)	4 (1.3%)
History of stroke/TIA, n (%)	59 (18.7%)
Beta-blockers on admission, n (%)	90 (28.5%)
RAAS inhibitors on admission, n (%)	120 (38.0%)
Antidepressants on admission, n (%)	12 (3.9%)
**HRV parameters**	
SDNN during daytime (ms), median (IQR)	35.5 (25–49)
RMSSD during daytime (ms), median (IQR)	20 (13–32)
LF during daytime (nu), median (IQR)	71.34 (52.8–83.14)
HF during daytime (nu), median (IQR)	21.02 (13.53–34.23)
LF/HF ratio during daytime, median (IQR)	3.28 (1.60–6.08)
SDNN during nighttime (ms), median (IQR)	37 (23–54)
RMSSD during nighttime (ms), median (IQR)	26 (17–39)
LF during nighttime (nu), median (IQR)	66.65 (46.96–81.79)
HF during nighttime (nu), median (IQR)	27.92 (15.92–43.27)
LF/HF ratio during nighttime, median (IQR)	2.30 (1.03–5.17)

*NIHSS, National Institute of Health Stroke Scale; TIA, transient ischemic attack; RAAS, renin angiotensin aldosteron system; HRV, heart rate variability; SDNN, standard deviation of beat-to-beat (NN) intervals; RMSSD, Root Mean Square of Successive Differences; LF, low frequency power component; HF, high frequency power component*.

The median time between onset of stroke-related symptoms and study related ECG recording was 57 h (95% CI 38–82 h) for chosen ECG dataset at daytime and 67 h (95% CI 47–92 h) for chosen ECG at dataset nighttime.

Age and NIHSS on admission were associated with reduced HRV. For full data on HRV parameters according to different baseline characteristics see Supplementary Tables 2A,B of the Supplementary Material.

### Clinical Outcome, Major Adverse Vascular Events or Death During Follow-Up

A mRS ≥ 2 was reported by 81 (23.7%) patients after three months and 80 patients (23.5%) after 12 months, respectively (see Supplementary Table 1 of Supplementary Material). Three months after the index stroke, 16 (4.5%) patients had suffered recurrent stroke, two (0.7%) had suffered MI and six patients had died (1.7%). The combined endpoint of adverse vascular events (recurrent stroke, MI and death) was reached by 21 patients (6.1%). After 12 months, 32 patients had suffered recurrent stroke (9.5%), five had suffered MI (1.7%) and 13 patients had died (3.7%). The combined endpoint of adverse vascular events (recurrent stroke, MI and death) was reached by 40 patients (12.2%).

Of the 13 patients who died after one year, four patients had died due to a malignant tumor, two had died after subarachnoid hemorrhage, one patient died after MI, one patient died due to heart failure and one died after recurrent ischemic stroke. The cause of death remained unknown in four patients. The frequency of clinical endpoints according to patients' baseline characteristics is shown in Supplementary Tables 3A,B of the Supplementary Material.

As shown in Tables 2, 3, we did not find any significant association between the any of the chosen HRV parameters and the occurrence of recurrent stroke or MI at three or 12 months after the index stroke, respectively.

**Table 2:** HRV and outcome at 90 days.

	**Death**	**Stroke**	**MI**	**MACE**	**mRS ≥ 2**
	**OR (95% CI),** ***p***	**OR (95% CI),** ***p***	**OR (95% CI),** ***p***	**OR (95% CI),** ***p***	**OR (95% CI),** ***p***
**Model 1**					
SDNN daytime	**0.846 (0.749–0.956)**, ***p*** = **0.008**	0.995 (0.965–1.026), *p* = 0.745	0.732 (0.462–1.159), *p* = 0.183	0.977 (0.947–1.008), *p* = 0.146	**0.982 (0.966–0.998)**, ***p*** = **0.029**
RMSSD daytime	**0.834 (0.705–0.986)**, ***p*** = **0.034**	0.985 (0.949–1.022), *p* = 0.408	0.674 (0.344–1.320), *p* = 0.25	0.966 (0.926–1.007), *p* = 0.103	0.993 (0.978–1.008), *p* = 0.353
SDNN nighttime	**0.899 (0.819–0.986)**, ***p*** = **0.024**	0.991 (0.969–1.015), *p* = 0.463	0.787 (0.55–1.128), *p* = 0.192	0.982 (0.959–1.006), *p* = 0.140	1.002 (0.992–1.011), *p* = 0.760
RMSSD nighttime	0.928 (0.85–1.013), *p* = 0.096	0.992 (0.968–1.016), *p* = 0.503	0.871 (0.653–1.162), *p* = 0.348	0.984 (0.958–1.011), *p* = 0.237	1.000 (0.991–1.009), *p* = 0.984
LF daytime	0.972 (0.938–1.007), *p* = 0.111	0.995 (0.971–1.019), *p* = 0.676	0.936 (0.849–1.031), *p* = 0.179	0.985 (0.965–1.006), *p* = 0.154	0.993 (0.981–1.005), *p* = 0.253
HF daytime	1.020 (0.976–1.065), *p* = 0.378	1.005 (0.975–1.036), *p* = 0.753	1.111 (0.973–1.267), *p* = 0.119	1.017 (0.991–1.043), *p* = 0.212	1.000 (0.985–1.016), *p* = 0.965
LF nighttime	0.995 (0.959–1.032), *p* = 0.775	0.991 (0.968–1.014), *p* = 0.424	0.968 (0.887–1.058), *p* = 0.447	0.992 (0.971–1.013), *p* = 0.445	0.993 (0.981–1.005), *p* = 0.228
HF nighttime	0.978 (0.931–1.027), *p* = 0.373	1.008 (0.981–1.036), *p* = 0.584	1.046 (0.941–1.163), *p* = 0.407	1.004 (0.980–1.029), *p* = 0.735	1.000 (0.987–1.014), *p* = 0.953
LF/HF daytime	0.762 (0.505–1.148), *p* = 0.193	0.917 (0.786–1.069), *p* = 0.266	0.067 (0–14.405), *p* = 0.325	0.876 (0.745–1.030), *p* = 0.109	1.004 (0.956–1.054), *p* = 0.886
LF/HF nighttime	1.025 (0.936–1.123), *p* = 0.593	0.893 (0.739–1.079), *p* = 0.239	0.394 (0.028–5.622), *p* = 0.492	0.969 (0.876–1.071), *p* = 0.539	1.015 (0.977–1.053), *p* = 0.449
**Model 2**					
SDNN daytime	**0.868 (0.769–0.979)**, ***p*** = **0.022**	n.a.	n.a.	n.a.	0.984 (0.967–1.0), *p* = 0.052
RMSSD daytime	1.014 (0.998–1.031), *p* = 0.093	n.a.	n.a.	n.a.	n.a.
SDNN nighttime	0.914 (0.833–1.004), *p* = 0.06	n.a.	n.a.	n.a.	n.a.
**Model 3**					
SDNN daytime	**0.851 (0.733–0.988)**, ***p*** = **0.034**	n.a.	n.a.	n.a.	n.a.

*Logistic regression analysis according to three different models. Model 1: unadjusted. Model 2: adjusted for age and sex. Model 3: adjusted for age, sex, NIHSS, diabetes, coronary artery disease, medication with beta-blockers, RAAS (renin-angiotensin-aldosterone system) inhibitors and antidepressants. HRV, heart rate variability; OR, odds ratio; mRS, modified Rankin scale; MI, myocardial infarction; MACE, major cardiovascular adverse event, combination of recurrent stroke, myocardial infarction and death; SDNN, standard deviation of beat-to-beat (NN) intervals; RMSSD, Root Mean Square of Successive Differences; LF, low frequency power component; HF, high frequency power component*.

*The bold values represent statistically significant results*.

**Table 3:** HRV and outcome at 365 days.

	**Death**	**Stroke**	**MI**	**MACE**	**mRS ≥ 2**
	**OR (95% CI),** ***p***	**OR (95% CI),** ***p***	**OR (95% CI),** ***p***	**OR (95% CI),** ***p***	**OR (95% CI),** ***p***
**Model 1**					
SDNN daytime	1.001 (0.971–1.033), *p* = 0.935	0.999 (0.978–1.021), *p* = 0.928	1.034 (0.984–1.086), *p* = 0.184	0.991 (0.971–1.012), *p* = 0.397	0.987 (0.973–1.001), *p* = 0.062
RMSSD daytime	**1.018 (1.001–1.035)**, ***p*** = **0.035**	1.000 (0.981–1.019), *p* = 0.983	**1.031 (1.009–1.054)**, ***p*** = **0.006**	1.000 (0.984–1.017), *p* = 0.969	0.993 (0.980–1.006), *p* = 0.291
SDNN nighttime	0.986 (0.960–1.013), *p* = 0.986	1.002 (0.988–1.016), *p* = 0.795	1.014 (0.983–1.047), *p* = 0.377	0.991 (0.976–1.07), *p* = 0.274	0.995 (0.986–1.005), *p* = 0.321
RMSSD nighttime	1.006 (0.990–1.022), *p* = 0.449	1.002 (0.990–1.015), *p* = 0.751	1.018 (0.998–1.038), *p* = 0.077	0.996 (0.982–1.010), *p* = 0.555	0.995 (0.986–1.004), *p* = 0.286
LF daytime	**0.964 (0.941–0.988)**, ***p*** = **0.004**	0.993 (0.975–1.010), *p* = 0.404	**0.946 (0.897–0.997)**, ***p*** = **0.039**	**0.983 (0.968–0.999)**, ***p*** = **0.038**	0.989 (0.979–1.000), *p* = 0.058
HF daytime	1.024 (0.994–1.055), *p* = 0.122	1.013 (0.991–1.035), *p* = 0.247	1.034 (0.977–1.095), *p* = 0.247	1.016 (0.996–1.036), *p* = 0.117	1.006 (0.992–1.020), *p* = 0.428
LF nighttime	0.978 (0.954–1.002), *p* = 0.076	1.00 (0.982–1.017), *p* = 0.969	0.985 (0.936–1.036), *p* = 0.555	0.994 (0.978–1.011), *p* = 0.486	0.993 (0.982–1.004), *p* = 0.192
HF nighttime	1.001 (0.971–1.031), *p* = 0.967	0.995 (0.975–1.016), *p* = 0.627	0.947 (0.868–1.034), *p* = 0.227	0.999 (0.980–1.019), *p* = 0.921	1.004 (0.991–1.017), *p* = 0.526
LF/HF daytime	0.808 (0.632–1.034), *p* = 0.091	0.889 (0.786–1.005), *p* = 0.06	0.588 (0.249–1.389), *p* = 0.226	**0.869 (0.769–0.982)**, ***p*** = **0.024**	0.977 (0.931–1.025), *p* = 0.348
LF/HF nighttime	0.967 (0.855–1.095), *p* = 0.6	1.009 (0.957–1.065), *p* = 0.73	0.941 (0.684–1.296), *p* = 0.711	1.011 (0.961–1.062), *p* = 0.678	0.975 (0.932–1.019), *p* = 0.258
**Model 2**					
RMSSD daytime	1.014 (0.998–1.031), *p* = 0.090	n.a.	**1.030 (1.005–1.055)**, ***p*** = **0.020**	n.a.	n.a.
LF daytime	**0.973 (0.948–0.999)**, ***p*** = **0.045**	n.a.	**0.943 (0.895–0.994)**, ***p*** = **0.03**	0.984 (0.967–1.000), *p* = 0.056	n.a.
LF/HF daytime	n.a.	n.a.	n.a.	**0.866 (0.763–0.984)**, ***p*** = **0.028**	n.a.
**Model 3**					
RMSSD daytime	n.a.	n.a.	1.033 (0.992–1.077), *p* = 0.119	n.a.	n.a.
LF daytime	0.972 (0.944–1.000), *p* = 0.053	n.a.	0.797 (0.578–1.098), *p* = 0.165	n.a.	n.a.
LF/HF daytime	n.a.	n.a.	n.a.	0.991 (0.890–1.103), *p* = 0.190	n.a.

*Logistic regression analysis according to three different models. Model 1: unadjusted. Model 2: adjusted for age and sex. Model 3: adjusted for age, sex, NIHSS, diabetes, coronary artery disease, medication with beta-blockers, RAAS (renin-angiotensin-aldosterone system) inhibitors and antidepressants. HRV, heart rate variability; OR, odds ratio; mRS, modified Rankin scale; MI, myocardial infarction; MACE, major cardiovascular adverse event, combination of recurrent stroke, myocardial infarction and death; SDNN, standard deviation of beat-to-beat (NN) intervals; RMSSD, Root Mean Square of Successive Differences; LF, low frequency power component; HF, high frequency power component*.

*The bold values represent statistically significant results*.

In the unadjusted analysis, SDNN and RMSSD at daytime as well as SDNN at nighttime were negatively associated with all-cause mortality after three months. This association remained significant for SDNN at daytime after full adjustment (OR 0.87, 95% CI 0.77–0.98, *p* = 0.022). In the unadjusted analysis, RMSSD and LF at daytime were associated with all-cause mortality after 12 months. This association was no longer observed after full adjustment.

We did not find any significant association between HRV parameters and poor functional outcome at three or 12 months after the index stroke, respectively.

For the subgroup analysis excluding patients with beta-blockers, RAAS inhibitors or antidepressants on admission, 160 patients remained (52% of the original study population). In this subgroup, the relative rate of major adverse vascular events was similar to the general study population (see Supplementary Table 4) except that no myocardial infarction occurred in this subgroup. In the subgroup analyses, we found a significant association between time domain HRV parameters during daytime and poor functional outcome. After adjustment, this association remained significant for SDNN and mRS ≥ 2 at 90 days as well as RMSSD and mRS ≥ 2 at 365 days (see Supplementary Tables 5A,B). In addition, we found a significant association between HF as well as LF/HF at daytime and the frequency of MACE and stroke at 365 days (see Supplementary Tables 5A,B).

## Discussion

Assessment of the impact of HRV on recurrent vascular events and clinical outcome after acute ischemic stroke was a pre-specified secondary aim of the HEBRAS study. To our knowledge, this is the first prospective study to examine whether there is a link between HRV in the acute phase of ischemic stroke and the prevalence of recurrent ischemic stroke or MI within 12 months.

In contrast to previous retrospective studies with smaller study populations and more severely affected stroke patients reporting an association between reduced HRV and mortality after stroke, we did not observe an association between HRV and mortality in patients with mild to moderate stroke severity after one year (5, 10, 17). However, we found a significant association between reduced HRV and mortality three months after stroke, based on time domain HRV parameters. The predictive value of HRV may have worn off over time. We do not have data on HRV in our patients at follow-up, so HRV may have gone back to normal, too. On the other hand, data suggest that HRV may stay impaired after stroke for a longer period of time (7).

In the subgroup of patients without medication with effect on the cardiovascular control (beta-blocker, RAAS inhibitors, antidepressants), we found indeed a significant association between time domain parameters and functional outcome on the one hand and HF (and subsequently the LF/HF ratio) and MACE as well as recurrent stroke after one year on the other hand. However, these effects were not detectable in the larger study population even after adjustment for medication on admission and should be interpreted with caution. This subgroup does not constitute the prespecified population and must therefore be considered as hypothesis generating. This finding may emphasize the impact of co-medication on HRV measurements nevertheless (18–20). Medication with effect on cardiovascular autonomic control may well-exhibit a protective effect. It is possible that the difference between the results of the whole study population and the results of the subgroup analysis are due to the effect of antihypertensive and antihypertensive medication, which cannot be fully controlled for by adjustment in statistical testing. On the other hand, those substances are frequently used in clinical routine. Indeed, almost half of our study population was excluded in the subgroup analysis without patients treated with beta-blockers, RAAS inhibitors or antidepressants. It is noteworthy that malignancy was the single most common reported cause of death in our study population and that only very few patients died from cardiac causes. HRV may therefore constitute a more general marker of reduced health and less specifically a marker for risk of vascular events.

Another possible explanation might be reduced statistical power of our analysis (type 2 error) due to the overall low mortality rate (1.7% after 90 days, 4.8% after one year) in our study population.

In addition, we were not able to show that HRV predicts functional outcome in stroke patients. This is inconsistent with other smaller studies (8, 9, 21), either using different scales (such as the Barthel Index or the Fugl-Meyer Scale) (9, 21) or a different mRS cut-off (8) to determine whether patients had good or poor functional outcome. Moreover, these studies examined more severely affected stroke patients. In our study, however, we included patients with mild to moderate stroke who had mostly excellent functional outcome (77% of patients had a mRS 0–1).

Strengths and limitations of the study have to be considered. HEBRAS is a prospective study with a predefined protocol and predefined endpoints that were published beforehand and registered on clinicaltrials.gov. However, as we included ischemic stroke patients only if they were able to provide informed consent, there was a considerable selection bias toward patients with mild to moderate stroke. This might have contributed to the fact that we observed a rather low number of cardiovascular adverse events at follow-up and that most patients had good or excellent functional outcome, further limiting the generalizability of the results. Despite frequent use, patient self-reported outcomes include a bias, too.

The ECG recordings we used for the calculation of HRV were taken from long-term Holter ECGs and were therefore not recorded under standardized conditions. On the other hand, the long-term ECG recording allowed us to examine HRV both during the day and at night, thus providing additional information on patients' autonomic cardiac function. As there is no universal recommendation which HRV parameters are best to examine the autonomic nervous system, the comparability of our data to previous studies is partly limited by the use of different HRV parameters.

## Conclusions

According to a predefined secondary analysis of a prospective study reduced HRV in the acute phase of mild to moderate ischemic stroke was not predictive of recurrent stroke or MI within one year. Co-medication effecting cardiovascular autonomic control may be taken into account in future studies. Larger prospective studies are needed to better understand the prognostic value of reduced HRV in stroke.

## Data Availability Statement

The raw data supporting the conclusions of this article will be made available by the authors, without undue reservation.

## Ethics Statement

The studies involving human participants were reviewed and approved by Ethics Committee of Charité-Universitätsmedizin Berlin. The patients/participants provided their written informed consent to participate in this study.

## Author Contributions

CN and KH conceived and designed the study. CN, KH, SH, JH, and TK acquired the data. RR, CN, JS, and KH conducted the analysis and interpretation of the data. All authors participated in drafting the paper or revising it critically and provided final approval.

## Funding

HEBRAS was funded by BMBF – CSB, grant G.2.17, Center for Stroke Research Berlin.

## Conflict of Interest

KH reports study grants by Bayer and Sanofi-Aventis, lecture fees/advisory board fees from Abbott, AstraZeneca, Bayer, Biotronik, Boehringer Ingelheim, Bristol-Myers-Squibb, Daiichi Sankyo, Edwards Lifesciences, Medtronic, Pfizer, Premier Research and Sanofi-Aventis, all outside the submitted work. ME reports grants from Bayer and fees paid to the Charité from AstraZeneca, Bayer, Boehringer Ingelheim, BMS, Daiichi Sankyo, Amgen, GSK, Sanofi, Covidien, Novartis, Pfizer, all outside the submitted work. CN received research grants from German Ministry of Research and Education, German Center for Neurodegenerative Diseases, German Center for Cardiovascular Research and also received speaker and/or consultation fees from Bayer, Boehringer Ingelheim, BMS, Daiichi Sankyo, Pfizer Pharma, Alexion, Abbott and W.L. Gore and Associates, all outside the submitted work. The remaining authors declare that the research was conducted in the absence of any commercial or financial relationships that could be construed as a potential conflict of interest.

## Publisher's Note

All claims expressed in this article are solely those of the authors and do not necessarily represent those of their affiliated organizations, or those of the publisher, the editors and the reviewers. Any product that may be evaluated in this article, or claim that may be made by its manufacturer, is not guaranteed or endorsed by the publisher.
