# Supplementary material for: Heart Rate Variability and Recurrent Stroke and Myocardial Infarction in Patients With Acute Mild to Moderate Stroke
Source: Front Neurol. 2021 Dec 23;12:772674. doi: 10.3389/fneur.2021.772674 (PMC8733333; doi:10.3389/fneur.2021.772674)
Supplement: Supplementary file 1 [file Table_1.DOCX]

Supplementary Material

**Table 1 Outcome of 308 HEBRAS patients included in the analysis.**

|  | 3 months after the index stroke | 12 months after the index stroke |
| --- | --- | --- |
| mRS ≥ 2, n (%) | 81 (23.7%) | 80 (23.5%) |
| death, n (%) | 6 (1.7%) | 13 (3.7%) |
| recurrent stroke, n (%) | 16 (4.5%) | 32 (9.5%) |
| MI, n (%) | 2 (0.7%) | 5 (1.7%) |
| MACE, n (%) | 21 (6.1%) | 40 (12.42%) |

**Abbreviations: mRS = modified Rankin scale, MI = myocardial infarction, MACE = major cardiovascular adverse event, combination of recurrent stroke, myocardial infarction and death**

**Table 2 Association between baseline characteristics and HRV parameters of 308 HEBRAS patients included in the analysis**

1. **Time domain parameters**

|  |  | SDNN daytime | RMSSD daytime | SDNN nighttime | RMSSD nighttime |
| --- | --- | --- | --- | --- | --- |
| Age |  | -0.252 (p<0.001) | -0.020 (p=0.714) | -0.186 (p=0.001) | -0.154 (p=0.005) |
| Sex | Male | 34 (25-51) | 19 (12-30) | 38.5 (23-57) | 26 (17-39) |
|  | Female | 35.5 (24-46) | 22 (14-33) | 33 (23-47) | 27 (17-41) |
|  | p | 0.492 | 0.233 | 0.087 | 0.407 |
| Initial NIHSS |  | -0.118 (p=0.033) | -0.021 (p=0.699) | -0.116 (p=0.035) | -0.037 (p=0.328) |
| History of hypertension | No | 36 (27-51) | 20 (12-32) | 40 (24-57) | 27 (17-43) |
|  | Yes | 33 (23-46) | 20 (13-32) | 36 (23-49) | 25 (17-38) |
|  | p | 0.177 | 0.576 | 0.082 | 0.293 |
| History of diabetes | No | 38 (27-50) | 21.5 (14-32) | 38 (25-55) | 27 (18-41) |
|  | Yes | 25.5 (19-40) | 15 (10-28) | 26 (17-48) | 17 (10-28) |
|  | p | < 0.001 | 0.002 | 0.001 | < 0.001 |
| History of coronary artery disease | No | 36 (25-49) | 21 (13-32) | 37.5 (24-54) | 27 (17.40) |
|  | Yes | 28 (23-41) | 18 (11-29) | 29 (20.5-55) | 20.5 (13-38) |
|  | p | 0.119 | 0.284 | 0.276 | 0.165 |
| History of heart failure | No | 35.5 (25-48) | 20 (13-32) | 37 (23-54) | 26 (17-39) |
|  | Yes | 22.5 (15-41.5) | 10 (9-33) | 27 (16.5-58.5) | 32 (17.5-68) |
|  | p | 0.079 | 0.067 | 0.380 | 0.373 |
| History of stroke/TIA | No | 36 (25-50) | 21 (13-34) | 28.5 (25-56) | 27 (17-42) |
|  | Yes | 27 (23-43.5) | 16.5 (11-24) | 27.5 (20-40) | 18.5 (14-28) |
|  | p | 0.015 | 0.003 | 0.002 | < 0.001 |
| Beta-blockers on admission | No | 37 (26-51) | 20.5 (13-32) | 38 (24-55) | 26 (17-40) |
|  | Yes | 29 (23-42) | 20 (12-30) | 33 (23-49.5) | 25 (16-38) |
|  | p | 0.003 | 0.879 | 0.080 | 0.472 |
| RAAS inhibitors on admission | No | 36 (26-52) | 21 (14-32) | 38 (24-58) | 27 (17-43) |
|  | Yes | 33 (23-43) | 19 (12-31.5) | 35.5 (22-49) | 23.5 (16-35) |
|  | p | 0.023 | 0.316 | 0.045 | 0.064 |
| Antidepressants on admission | No | 34 (24-48) | 20 (12-31) | 37 (23-54) | 26 (17-39) |
|  | Yes | 33 (28-51) | 24 (15-51) | 30 (23-55) | 25 (18-45) |
|  | p | 0.789 | 0.286 | 0.696 | 0.745 |

**Median (IQR) of time domain parameters according to baseline characteristics. Univariate analyses using Mann-Whitney U test for binomially scaled and Correlation analysis for continuously scaled baseline characteristics. Abbreviations: HRV = heart rate variability. NIHSS = National institute of health stroke scale, TIA = transient ischemic attack, RAAS = renin angiotensin aldosterone system, SDNN = standard deviation of beat-to-beat (NN) intervals, RMSSD = Root Mean Square of Successive Differences**

1. **Frequency domain parameters**

|  |  | LF daytime | HF daytime | LF nighttime | HF nighttime | LF/HF daytime | LF/HF nighttime |
| --- | --- | --- | --- | --- | --- | --- | --- |
| Age |  | -0.312 (p<0.001) | 0.216 (p<0.001) | 0.06 (p=0.280) | -0.145 (p=0.009) | -0.255 (p<0.001) | 0.086 (p=0.119) |
| Sex | Male | 76.07 (57.09-85.11) | 19.5 (11.66-29.74) | 69.43 (55.24-83.45) | 26.37 (14.05-39.9) | 3.92 (1.96-7.34) | 2.58 (1.39-5.96) |
|  | Fe-male | 64.58 (43.51-78.33) | 25.4 (15.67-41.63) | 59.16 (38.97-73.14) | 34.78 (22.03-51.76) | 2.31 (1.07-4.92) | 1.7 (0.69-3.17) |
|  | p | < 0.001 | 0.001 | < 0.001 | 0.001 | <0.001 | <0.001 |
| Initial NIHSS |  | -0.169 (p=0.002) | 0.104 (p=0.061) | -0.039 (p=0.377) | 0.001 (p=0.98) | -0.129 (p=0.02) | -0.025 (p=0.650) |
| History of hyper-tension | No | 77.19 (61.54-86.44) | 18.7 (9.68-28.37) | 66.74 (45.4-82.86) | 27.56 (14.65-44.5) | 4.14 (2.11-8.78) | 2.36 (1.05-5.69) |
|  | yes | 67.26 (46.56-80.86) | 22.87 (15.22-41) | 65.74 (46.23-80.26) | 28.72 (16.23-46.6) | 2.72 (1.19-5.1) | 2.24 (0.97-4.82) |
|  | p | < 0.001 | < 0.001 | 0.601 | 0.734 | < 0.001 | 0.681 |
| History of diabetes | No | 73.31 (53.34-84.03) | 21.01 (12.54-34.25) | 65.85 (45.01-81.68) | 29.13 (16.02-46.78) | 3.78 (1.61-3.48) | 2.24 (0.92-5.13) |
|  | yes | 66.01 (45.74-77.88) | 22.37 (15.73-35.82) | 67.25 (55.38-67.25) | 26.74 (15.21-38.51) | 2.73 (1.38-4.91) | 2.44 (1.43-5.39) |
|  | P | 0.013 | 0.193 | 0.429 | 0.130 | 0.055 | 0.280 |
| History of coronary artery disease | No | 71.44 (51.17-83.11) | 21.01 (13.45-34.26) | 66.07 (46.41-81.61) | 28.62 (15.94-28.62) | 3.31 (1.58-6.14) | 2.28 (1.03-5.17) |
|  | yes | 65.19 (55.42-78.52) | 22.87 (15.43-36.2) | 66.92 (37.9-81.77) | 26.74 (15.94-47.83) | 2.64 (1.49-5.09) | 2.46 (0.8-5.11) |
|  | p | 0.360 | 0.396 | 0.905 | 0.618 | 0.416 | 0.785 |
| History of heart failure | No | 71.22 (52-83.08) | 21.02 (13.69-34.53) | 66.19 (46.96-81.84) | 28.23 (15.93-43.42) | 3.27 (1.54-6.01) | 2.28 (1.03-5.14) |
|  | yes | 67.7 (51.74-73.77) | 23.47 (18.56-40.44) | 35.62 (10.69-72.58) | 47.04 (19.08-66.4) | 2.89 (1.5-4.15) | 0.76 (0.16-4.73) |
|  | p | 0.364 | 0.447 | 0.074 | 0.231 | 0.476 | 0.145 |
| History of stroke/ TIA | No | 70.61 (50.75-70.61) | 21.26 (13.53-34.67) | 66.1 (45.08-81.61) | 28.16 (16.04-46.72) | 3.14 (1.49-5.95) | 2.28 (0.93-5.14) |
|  | yes | 72.72 (56-62-82.25) | 20.59 (13.81-29.52) | 66.14 (55.8-81.86) | 28.72 (15.39-40.93) | 3.4 (2.08-5.94) | 2.26 (1.36-5.36) |
|  | p | 0.868 | 0.789 | 0.463 | 0.548 | 0.734 | 0.469 |
| Beta-blockers on admission | No | 74.42 (57.71-85.81) | 19.5 (11.01-30.46) | 66.62 (46.23-82.19) | 27.48 (15.69-44.47) | 3.87 (1.86-7.5) | 2.39 (1.03-5.24) |
|  | yes | 63.64 (44.11-77.73) | 26.17 (17.4-43.5) | 63.72 (45.6-78.4) | 29.7 (17.12-29.7) | 2.23 (0.98-4.6) | 2.02 (0.94-4.43) |
|  | p | < 0.001 | < 0.001 | 0.206 | 0.477 | < 0.001 | 0.266 |
| RAAS inhibitors on admission | No | 75.83 (56.27-85.03) | 19.1 (12.22-30.35) | 66.41 (46.41-82.52) | 28.25 (15.12-45.15) | 3.93 (1.88-7.01) | 2.3 (0.98-5.48) |
|  | yes | 65.19 (47.36-79.01) | 24.41 (15.84-42.3) | 65.75 (46.08-78.27) | 28.28 (17.68-43.13) | 2.68 (1.16-4.87) | 2.21 (0.99-4.4) |
|  | p | 0,.001 | 0.001 | 0.624 | 0.753 | 0.001 | 0.615 |
| Antide-pressants on admission | No | 71.42 (53.15-83.22) | 21.01 (13.46-34.22) | 66.11 (46.41-81.79) | 28.11 (15.92-44.21) | 3.29 (1.61-6.18) | 2.28 (1.03-5.17) |
|  | yes | 59.27 (30.01-79.43) | 20.46 (14.31-55.67) | 69.58 (30.51-87.42) | 26.83 (11.18-51.73) | 2.11 (0.75-5.18) | 2.65 (0.49-7.81) |
|  | p | 0.051 | 0.499 | 0.987 | 0.881 | 0.204 | 0.956 |

**Median (IQR) of time domain parameters according to baseline characteristics. Univariate analyses using Mann-Whitney U test for binomially scaled and Correlation analysis for continuously scaled baseline characteristics. Abbreviations: HRV = heart rate variability, NIHSS = National institute of health stroke scale, TIA = transient ischemic attack, RAAS = renin angiotensin aldosterone system, LF = low frequency power component, HF = high frequency power component**

**Table 3 Association between baseline characteristics and outcome of 308 HEBRAS patients included in the analysis**

1. **at 90 days**

|  | Total population | Death (n=6) | Stroke (n=16) | MI (n=2) | MACE (n=21) | mRS ≥ 2 (n=81) |
| --- | --- | --- | --- | --- | --- | --- |
| Age, median (IQR) | 69 (58-75) | 74.5 (72.25-80.5), p=0.034 | 68.5 (60.5-74.75), p=0.867 | 73-80, p=0,110 | 72 (65.5-76), p=0.168 | 71 (59-75.5), p=0.057 |
| Sex, n | Male (n=191) | 3 | 11 | 0 | 11 | 53 |
|  | Female (n=117) | 3 | 5 | 2 | 10 | 28 |
|  | p | 0.675 | 0.985 | 0.142 | 0.334 | 0.465 |
| Initial NIHSS, median (IQR) | 2 (1-4) | 3.5 (2-6), p=0.135 | 1.5 (0-3), p=0.174 | 0-2, p=0.786 | 2 (0-4), p=0.359 | 3 (2-6), p < 0.001 |
| History of hypertension, n | No (n=125) | 1 | 11 | 0 | 11 | 35 |
|  | Yes (n=183) | 5 | 5 | 2 | 10 | 46 |
|  | p | 0.242 | 0.040 | 0.508 | 0.369 | 0.962 |
| History of diabetes, n | No (n=244) | 3 | 14 | 1 | 16 | 64 |
|  | Yes (n=64) | 3 | 2 | 1 | 5 | 17 |
|  | p | 0.088 | 0.746 | 0.347 | 0.589 | 0.659 |
| History of coronary artery disease, n | No (n=278) | 5 | 16 | 2 | 20 | 77 |
|  | Yes (n=30) | 1 | 0 | 0 | 1 | 4 |
|  | p | 0.448 | 0.380 | 1.0 | 0.706 | 0.132 |
| History of heart failure, n | No (n=304) | 6 | 16 | 2 | 21 | 78 |
|  | Yes (n=4) | 0 | 0 | 0 | 0 | 3 |
|  | p | 1.0 | 1.0 | 1.0 | 1.0 | 0.147 |
| History of stroke/TIA, n | No (n=249) | 5 | 13 | 2 | 18 | 65 |
|  | Yes (n=59) | 1 | 3 | 0 | 3 | 16 |
|  | p | 1.0 | 1.0 | 1.0 | 1.0 | 0.606 |
| Beta-blockers on admission, n | No (n=218) | 4 | 15 | 1 | 17 | 55 |
|  | Yes (n=90) | 2 | 1 | 1 | 4 | 25 |
|  | p | 0.675 | 0.048 | 0.486 | 0.455 | 0.525 |
| RAAS inhibitors on admission, n | No (n=188) | 3 | 14 | 0 | 14 | 49 |
|  | Yes (n=120) | 3 | 2 | 2 | 7 | 32 |
|  | p | 0.674 | 0.036 | 0.138 | 0.689 | 0.658 |
| Antidepressants on admission, n | No (n=296) | 6 | 16 | 2 | 21 | 77 |
|  | Yes (n=12) | 0 | 0 | 0 | 0 | 4 |
|  | p | 1.0 | 1.0 | 1.0 | 1.0 | 0.492 |

**Frequency of clinical outcome events according to baseline characteristics. Univariate analyses using using Chi-Square test/Fisher’s exact test for binomial and Mann-Whitney U test for continuous variables. Abbreviations: NIHSS = National institute of health stroke scale, TIA = transient ischemic attack, RAAS = renin angiotensin aldosterone system**

1. **at 365 days**

|  | Total population | Death (n=13) | Stroke (n=30) | MI (n=5) | MACE (n=40) | mRS ≥ 2 (n=80) |
| --- | --- | --- | --- | --- | --- | --- |
| Age, median (IQR) | 69 (58-75) | 74 (71-78.5), p=0.004 | 70.5 (58.75-74.25), p=0.998 | 73 (63-83.5), p=0.244 | 71.5 (65-75), p=0.330 | 70 (58-75), p=0.750 |
| Sex, n | Male (n=191) | 7 | 20 | 3 | 25 | 52 |
|  | Female (n=117) | 6 | 10 | 2 | 15 | 28 |
|  | p | 0.519 | 0.550 | 0.293 | 0.965 | 0.339 |
| Initial NIHSS, median (IQR) | 2 (1-4) | 2 (1.5-4), p=0.315 | 2 (0-4), p=0.808 | 4 (2-9.5), p=0.100 | 2 (0-4), p=0.525 | 3 (1.5-5), p < 0.001 |
| History of hypertension, n | No (n=125) | 3 | 15 | 0 | 16 | 30 |
|  | Yes (n=183) | 10 | 15 | 5 | 24 | 50 |
|  | p | 0.252 | 0.618 | 0.266 | 0.830 | 0.555 |
| History of diabetes, n | No (n=244) | 6 | 24 | 1 | 28 | 56 |
|  | Yes (n=64) | 7 | 6 | 4 | 12 | 24 |
|  | p | 0.002 | 1.0 | 0.006 | 0.80 | 0.013 |
| History of coronary artery disease, n | No (n=278) | 11 | 28 | 4 | 36 | 72 |
|  | Yes (n=30) | 2 | 2 | 1 | 4 | 8 |
|  | p | 0.354 | 1.0 | 0.389 | 0.778 | 0.722 |
| History of heart failure, n | No (n=304) | 12 | 28 | 5 | 38 | 77 |
|  | Yes (n=4) | 1 | 2 | 0 | 2 | 3 |
|  | p | 0.206 | 0.099 | 1.0 | 0.165 | 0.207 |
| History of stroke/TIA, n | No (n=249) | 11 | 22 | 5 | 31 | 61 |
|  | Yes (n=59) | 2 | 8 | 0 | 9 | 19 |
|  | p | 1.0 | 0.235 | 0.588 | 0.516 | 0.414 |
| Beta-blockers on admission, n | No (n=218) | 10 | 22 | 1 | 26 | 55 |
|  | Yes (n=90) | 3 | 8 | 4 | 14 | 25 |
|  | p | 1.0 | 0.749 | 0.027 | 0.412 | 0.882 |
| RAAS inhibitors on admission, n | No (n=188) | 7 | 21 | 0 | 23 | 45 |
|  | Yes (n=120) | 6 | 9 | 5 | 17 | 35 |
|  | p | 0.519 | 0.321 | 0.007 | 0.543 | 0.588 |
| Antidepressants on admission, n | No (n=296) | 12 | 30 | 5 | 39 | 76 |
|  | Yes (n=12) | 1 | 0 | 0 | 1 | 4 |
|  | p | 0.376 | 0.612 | 1.0 | 1.0 | 0.760 |

**Frequency of clinical outcome events according to baseline characteristics. Univariate analyses using using Chi-Square test/Fisher’s exact test for binomial and Mann-Whitney U test for continuous variables. Abbreviations: NIHSS = National institute of health stroke scale, TIA = transient ischemic attack, RAAS = renin angiotensin aldosterone system**

**Table 4 Outcome of 160 HEBRAS patients without beta-blockers, RAAS inhibitors or antidepressants at baseline**

|  | 3 months after the index stroke | 12 months after the index stroke |
| --- | --- | --- |
| mRS ≥ 2, n (%) | 34 (21.3%) | 49 (30.6%) |
| death, n (%) | 3 (1.9%) | 6 (3.8%) |
| recurrent stroke, n (%) | 13 (8.1%) | 17 (10.6%) |
| MI, n (%) | 0 (0.0%) | 0 (0.0%) |
| MACE, n (%) | 13 (8.1%) | 18 (11.3%) |

**Abbreviations: RAAS = renin angiotensin aldosterone system, mRS = modified Rankin scale, MI = myocardial infarction, MACE = major cardiovascular adverse event, combination of recurrent stroke, myocardial infarction and death**

**Table 5 HRV and Outcome of 160 HEBRAS patients without beta-blockers, RAAS inhibitors or antidepressants at baseline**

1. **at 90 days**

| **Model 1** | | | | |
| --- | --- | --- | --- | --- |
|  | **Death** | **Stroke** | **MACE** | **mRS ≥ 2** |
|  | **OR (95% CI), p** | **OR (95% CI), p** | **OR (95% CI), p** | **OR (95% CI), p** |
| SDNN daytime | 0.881 (0.767-1.012), p=0.074 | 0.990 (0.959-1.022), p=0.533 | 0.990 (0.959-1.022), p=0.533 | ***0.970 (0.948-0.993), p=0.012*** |
| RMSSD daytime | 0.911 (0.782-1.062), p=0.233 | 0.989 (0.951-1.029), p=0.599 | 0.989 (0.951-1.029), p=0.599 | ***0.967 (0.937-0.997), p=0.034*** |
| SDNN nighttime | 0.872 (0.743-1.023), p=0.092 | 0.992 (0.969-1.017), p=0.532 | 0.992 (0.969-1.017), p=0.532 | 0.997 (0.983-1.012), p=0.725 |
| RMSSD nighttime | 0.935 (0.832-1.052), p=0.263 | 0.992 (0.967-1.018), p=0.546 | 0.992 (0.967-1.018), p=0.546 | 0.990 (0.973-1.007), p=0.250 |
| LF daytime | 0.972 (0.923-1.024), p=0.972 | 0.980 (0.953-1.007), p=0.141 | 0.980 (0.953-1.007), p=0.141 | 0.995 (0.976-1.015), p=0.644 |
| HF daytime | 1.018 (0.952-1.087), p=0.607 | 1.026 (0.993-1.059), p=0.125 | 1.026 (0.993-1.059), p=0.125 | 0.998 (0.972-1.023), p=0.854 |
| LF nighttime | 0.979 (0.930-1.032), p=0.434 | 0.986 (0.961-1.011), p=0.276 | 0.986 (0.961-1.011), p=0.276 | 1.001 (0.983-1.019), p=0.942 |
| HF nighttime | 0.989 (0.938-1.054), p=0.730 | 1.010 (0.982-1.038), p=0.506 | 1.010 (0.982-1.038), p=0.506 | 0.990 (0.970-1.011), p=0.345 |
| LF/HF daytime | 0.773 (0.467-1.281), p=0.318 | 0.837 (0.687-1.018), p=0.076 | 0.837 (0.687-1.018), p=0.076 | 1.005 (0.942-1.073), p=0.871 |
| LF/HF nighttime | 0.847 (0.521-1.379), p=0.504 | 0.878 (0.717-1.076), p=0.211 | 0.878 (0.717-1.076), p=0.211 | 1.053 (0.992-1.118), p=0.088 |
| **Model 2** | | | | |
|  | **Death** | **Stroke** | **MACE** | **mRS ≥ 2** |
|  | **OR (95% CI), p** | **OR (95% CI), p** | **OR (95% CI), p** | **OR (95% CI), p** |
| SDNN daytime | n.a. | n.a. | n.a. | ***0.971 (0.947-0.995), p=0.017*** |
| RMSSD daytime | n.a. | n.a. | n.a. | ***0.967 (0.937-0.999), p=0.043*** |
| **Model 3** | | | | |
|  | **Death** | **Stroke** | **MACE** | **mRS ≥ 2** |
|  | **OR (95% CI), p** | **OR (95% CI), p** | **OR (95% CI), p** | **OR (95% CI), p** |
| SDNN daytime | n.a. | n.a. | n.a. | ***0.973 (0.947-0.999), p=0.043*** |
| RMSSD daytime | n.a. | n.a. | n.a. | 0.966 (0.932-1.002), p=0.064 |

**Logistic regression analysis according to three different models. Model 1: unadjusted. Model 2: adjusted for age and sex. Model 3: adjusted for age, sex, NIHSS, diabetes and coronary artery disease. Abbreviations: HRV = heart rate variability, RAAS = renin angiotensin aldosterone system, OR = odds ratio, mRS = modified Rankin scale, MACE = major cardiovascular adverse event, combination of recurrent stroke, myocardial infarction and death, SDNN = standard deviation of beat-to-beat (NN) intervals, RMSSD = Root Mean Square of Successive Differences, LF = low frequency power component, HF = high frequency power component**

1. **at 365 days**

| **Model 1** | | | | |
| --- | --- | --- | --- | --- |
|  | **Death** | **Stroke** | **MACE** | **mRS ≥ 2** |
|  | **OR (95% CI), p** | **OR (95% CI), p** | **OR (95% CI), p** | **OR (95% CI), p** |
| SDNN daytime | ***0.874 (0.788-0.969), p=0.011*** | 0.993 (0.965-1.020), p=0.595 | 0.989 (0.962-1.016), p=0.413 | ***0.978 (0.959-0.997), p=0.023*** |
| RMSSD daytime | 0.940 (0.860-1.027), p=0.170 | 0.991 (0.958-1.025), p=0.608 | 0.990 (0.958-1.024), p=0.570 | ***0.970 (0.944-0.996), p=0.022*** |
| SDNN nighttime | 0.940 (0.880-1.004), p=0.066 | 0.992 (0.970-1.015), p=0.486 | 0.992 (0.969-1.017), p=0.532 | 1.0 (0.987-1.013), p=0.980 |
| RMSSD nighttime | 0.962 (0.904-1.024), p=0.225 | 0.992 (0.968-1.015), p=0.481 | 0.993 (0.971-1.015), p=0.544 | 0.994 (0.980-1.008), p=0.416 |
| LF daytime | ***0.955 (0.918-0.993), p=0.022*** | 0.977 (0.953-1.001), p=0.064 | ***0.973 (0.950-0.997), p=0.030*** | 0.986 (0.970-1.004), p=0.119 |
| HF daytime | 1.041 (0.997-1.086), p=0.068 | ***1.036 (1.006-1.067), p=0.019*** | ***1.039 (1.009-1.069), p=0.011*** | 1.010 (0.988-1.032), p=0.374 |
| LF nighttime | ***0.958 (0.920-0.998), p=0.042*** | 0.982 (0.959-1.006), p=0.143 | 0.981 (0.959-1.004), p=0.109 | 0.993 (0.978-1.009), p=0.404 |
| HF nighttime | 1.027 (0.987-1.068), p=0.196 | 1.017 (0.991-1.043), p=0.211 | 1.018 (0.993-1.044), p=0.162 | 1.004 (0.986-1.021), p=0.685 |
| LF/HF daytime | 0.724 (0.476-1.102), p=0.132 | ***0.814 (0.676-0.981), p=0.030*** | ***0.797 (0.658-0.965), p=0.020*** | 0.960 (0.898-1.027), p=0.235 |
| LF/HF nighttime | 0.647 (0.350-1.198), p=0.166 | 0.857 (0.701-1.048), p=0.133 | 0.845 (0.686-1.040), p=0.112 | 0.993 (0.934-1.056), p=0.835 |
| **Model 2** | | | | |
|  | **Death** | **Stroke** | **MACE** | **mRS ≥ 2** |
|  | **OR (95% CI), p** | **OR (95% CI), p** | **OR (95% CI), p** | **OR (95% CI), p** |
| SDNN daytime | ***0.891 (0.805-0.986), p=0.026*** | n.a. | n.a. | ***0.976 (0.956-0.996), p=0.019*** |
| RMSSD daytime | n.a. | n.a. | n.a. | ***0.968 (0.942-0.995), p=0.020*** |
| LF daytime | 0.957 (0.915-1.000), p=0.052 | n.a. | ***0.974 (0.950-0.998), p=0.036*** | n.a. |
| HF daytime | n.a. | ***1.037 (1.006-1.068), p=0.018*** | ***1.039 (1.009-1.070), p=0.011*** | n.a. |
| LF nighttime | ***0.954 (0.913-0.996), p=0.032*** | n.a. | n.a. | n.a. |
| LF/HF daytime | n.a. | ***0.806 (0.667-0.976), p=0.027*** | ***0.794 (0.653-0.965), p=0.020*** | n.a. |
| **Model 3** | | | | |
|  | **Death** | **Stroke** | **MACE** | **mRS ≥ 2** |
|  | **OR (95% CI), p** | **OR (95% CI), p** | **OR (95% CI), p** | **OR (95% CI), p** |
| SDNN daytime | 0.903 (0.814-1.0), p=0.051 | n.a. | n.a. | 0.979 (0.958-1.0), p=0.052 |
| RMSSD daytime | n.a. | n.a. | n.a. | ***0.969 (0.941-0.997), p=0.033*** |
| LF daytime | n.a. | n.a. | 0.974 (0.950-1.0), p=0.050 | n.a. |
| HF daytime | n.a. | ***1.036 (1.006-1.068), p=0.020*** | ***1.038 (1.008-1.069), p=0.012*** | n.a. |
| LF nighttime | ***0.954 (0.912-0.998), p=0.039*** | n.a. | n.a. | n.a. |
| LF/HF daytime | n.a. | ***0.811 (0.672-0.979), p=0.029*** | ***0.799 (0.659-0.968), p=0.022*** | n.a. |

**Logistic regression analysis according to three different models. Model 1: unadjusted. Model 2: adjusted for age and sex. Model 3: adjusted for age, sex, NIHSS, diabetes and coronary artery disease. Abbreviations: HRV = heart rate variability, RAAS = renin angiotensin aldosterone system, OR = odds ratio, mRS = modified Rankin scale, MACE = major cardiovascular adverse event, combination of recurrent stroke, myocardial infarction and death, SDNN = standard deviation of beat-to-beat (NN) intervals, RMSSD = Root Mean Square of Successive Differences, LF = low frequency power component, HF = high frequency power component**
